# Inadvertent arterial puncture involving the subclavian artery and the aorta during central venous catheterization: a case report

**DOI:** 10.1186/s13256-021-02871-w

**Published:** 2021-05-28

**Authors:** Mark Henry Alon

**Affiliations:** Department of Medicine, Division of Hospital Medicine, Mayo Clinic Health Systems in Affiliation with Mayo Clinic College of Medicine and Science, 1221 Whipple Street, Eau Claire, WI 5470 USA

**Keywords:** Subclavian catheterization, Central venous catherization, Arterial puncture, Subclavian artery, Aorta, Case report

## Abstract

**Background:**

This case report describes a subclavian vein cannulation that inadvertently led to an arterial puncture with the catheter tip radiologically seen at the level of the aorta. This case emphasizes the importance of postprocedural imaging and the disadvantages of not using ultrasound guidance in central venous catheterization.

**Case presentation:**

A 24-year-old Caucasian man with diabetes mellitus type 1 presented himself to the emergency department due to abdominal pain accompanied by nausea and vomiting. The patient’s vital signs revealed blood pressure of 84/53 mmHg, heart rate of 103 beats per minute, respiratory rate of 18 breaths per minute, and temperature of 98.2 °F (36.7 °C). On physical examination, he was found to have dry oral mucosa with poor skin turgor, with diagnostics showing that he was in diabetic ketoacidosis after running out of insulin for 2 days. The patient was transferred to the intensive care unit to receive a higher level of care. Unfortunately, due to difficulty of peripheral line placement, only a gauge-22 cannula was secured at the left dorsum of the hand. Efforts to replace the current peripheral line were unsuccessful, and a decision to perform a central vein cannulation via the internal jugular vein was made. This was futile as well due to volume depletion, prompting a subsequent right subclavian vein route attempt. The procedure inadvertently punctured the arterial circulation, leading to the catheter tip being visible at the level of the aorta on postprocedure X-ray. The subclavian line was immediately removed with no adverse consequences for the patient. A right femoral line was successfully placed, and continuous management of the diabetic ketoacidosis ensued until normalization of the high anion gap was achieved.

**Conclusion:**

Utilization of real-time ultrasound guidance via the subclavian approach could have allowed for direct visualization of needle insertion to the anatomical structures, guidewire location, and directionality, all of which can lead to decreased complications and improved cannulation success compared with the landmark technique. A leftward direction of the catheter seen on postprocedural X-rays should raise high suspicion of inadvertent catheter placement and immediate correction. This complication should have been prevented if ultrasound guidance had been used.

## Background

In the USA alone, more than 5 million central venous catheters are placed yearly for hemodynamic monitoring, medication delivery, and nutritional support. However, procedural complications during the insertion of central line catheters occur at approximately 15% [1]. Subclavian catheterization is often uncomplicated and successful but with reported complication rates of between 0.3% and 12%, according to the experience of the physician and type of complication [2]. In particular, inadvertent arterial puncture carries a 3.7% risk [3] and can cause significant morbidity and mortality if not recognized early.

From the year 2001 to 2004, multiple prospective, randomized trials and metaanalyses addressing real-time ultrasound guidance in central venous catheter placement consistently demonstrated lower rates of complication, shorter procedure times, and higher success rates. Considering these studies, its adoption as standard of care has been recommended by several healthcare organizations, including the Centers for Disease Control and Prevention, Institute of Medicine, National Institute for Health and Care Excellence, Agency for Healthcare Research and Quality, and several medical specialty societies [1]. Despite numerous literature studied demonstrating fewer complications for ultrasound-guided central venous catheterization, it is not universally utilized in subclavian central line catheterization compared with internal jugular and femoral line catheterization, respectively. We present a case report wherein subclavian vein cannulation lead to arterial puncture, with the catheter tip radiologically seen at the level of the aorta.

## Case presentation

A 24-year-old, 165-lb male Caucasian patient with diabetes mellitus type 1 presented to the emergency department with a complaint of abdominal pain accompanied by nausea and vomiting. The patient stated that he was unable to self-administer basal insulin for two days after he ran out of insulin pen needles. His vital signs on presentation revealed blood pressure of 84/53 mmHg, heart rate of 103 beats per minute, respiratory rate of 18 breaths per minute, and temperature of 98.2 °F (36.7 °C). Physical examination at time of presentation showed that he was ill-looking, with dry oral mucosa and poor skin turgor. Initial diagnostic tests showed high anion gap, metabolic acidosis, ketonemia, and hyperglycemia, prompting the start of diabetic ketoacidosis management and subsequent intensive care unit (ICU) transfer (Table 1). At this time, the patient only had a gauge-22 peripheral line placed at the dorsum of his left hand after multiple unsuccessful attempts at placing a larger peripheral line gauge.

**Table 1:** Initial laboratory results showing diabetic ketoacidosis

Arterial blood gas		Chemistry		Urinalysis	
pH	7.243	Na	124 mmol/L	Color	Straw
paCO_2_	20.3	K	5.51 mmol/L	Appearance	Clear
paO_2_	118	Cl	88 mmol/L	Glucose	3+
sO_2_	98.4	HCO_3_	9 mmol/L	Urine ketones	3+
cHCO_3_	8.4	BUN	19 mg/dL	Specific gravity	1.02
Lactate	1	Creatinine	1.6 mg/dL	Urine pH	5.5
		Glucose	632 mg/dL		
		Calcium	9.1 mg/dL		

**Fig. 1:**
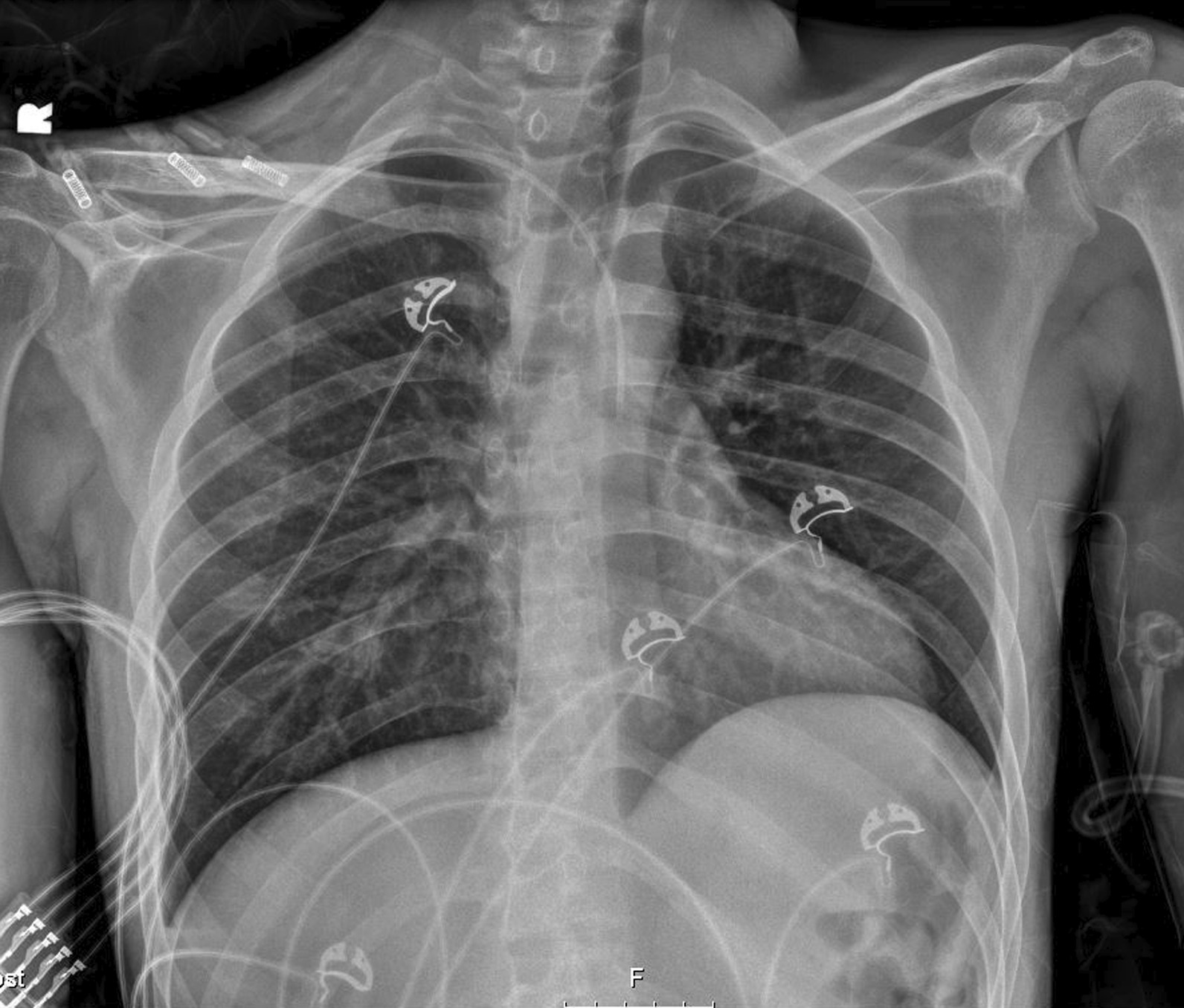
Central line cannulation via subclavian vessel with tip at aorta

**Fig. 2:**
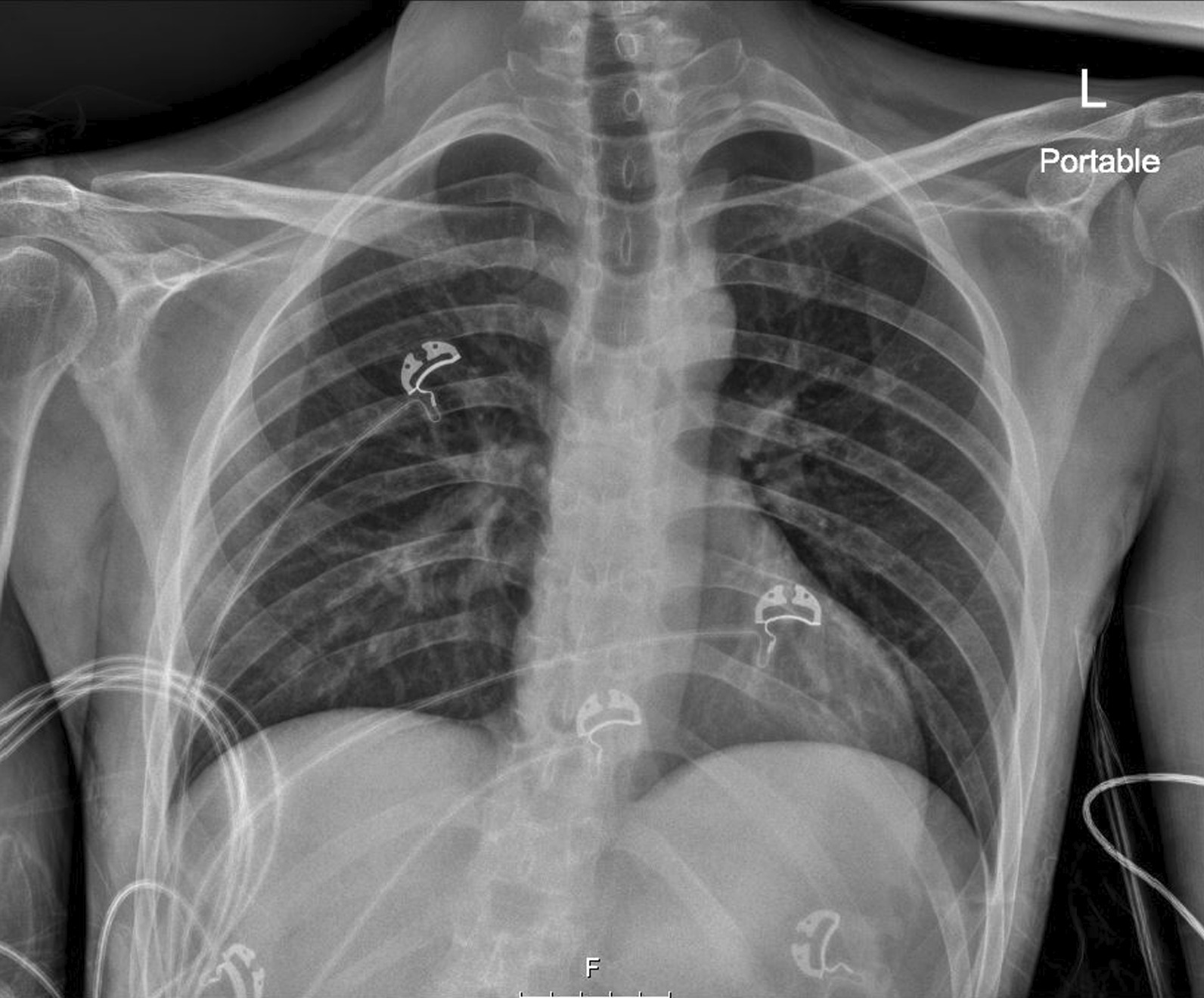
Chest radiograph showing absence of serious complication after prompt removal

In the ICU, efforts at internal jugular vein central line placement were unsuccessful as well due to the patient’s low volume status, making the target vein extremely difficult to cannulate even by increasing Trendelenburg tilt and doing venipuncture at end inspiration. Right subclavian vein catheterization using the infraclavicular approach was instead performed. However, the postprocedure chest X-ray showed that the catheter was somehow in the aorta (Fig. 1). Blood gas obtained from the subclavian central line revealed arterial placement, prompting removal of the catheter with no serious consequences to the patient (Table 2; Fig. 2). A right femoral line was successfully placed, and continuous management of diabetic ketoacidosis was achieved with eventual normalization of the anion gap and clinical improvement of the patient. The patient stayed in the intensive care unit for 2 days and was then transferred to the medicine floors for continuation of glucose management with eventual discharge on the 4th day after case management; social work assisted in securing his insulin supplies, and diabetic education was performed.

**Table 2:** Arterial blood gas result confirming that the catheter was in the arterial vessel

Arterial blood gas	
pH	7.252
paCO_2_	23.2
paO_2_	112
sO_2_	98
cHCO_3_	10.3
Lactate	0.79

## Discussion

More than 5 million central venous catheters are placed per year in the USA alone [4], playing an essential role in hemodynamic monitoring, medication and fluid delivery, and nutritional support in the intensive care unit. Central venous catheterization is dependent on the use of standard techniques and the skill of the clinician, with complication rates being reported at 15% [1].

The central venous catheterization approach using the subclavian vein route was first described by Aubaniac in 1952 [5, 5] and became popular after Wilson *et al.* in 1962 described its use in central venous pressure (CVP) measurement [7]. As widespread use increased, so did complications related to catheterization, which include pneumothorax, hemothorax, hydrothorax, chylothorax, catheter emboli, brachial plexus injury, perforation of vein or heart, sepsis, thrombophlebitis, air embolus, and hydromediastinum [8].

A study analyzing the catheter tip location showed that, 71% of the time, the catheter tip will be located in the superior vena cava or innominate vein, and that 80.1% of left-sided catheters were positioned properly versus 63.2% of right-sided catheters. A 21.4% risk is present in placing a subclavian catheter in the right atrium, with right atrial placement being more common, with right-sided catheter at 29.4% compared with 12.1% with left-sided catheters. The remainder were located in the jugular vein (4.6%), the opposite innominate vein (1.4%), subclavian vein (0.6%), right ventricle (0.4%), opposite subclavian vein (0.2%), inferior vena cava (0.2%), or left-sided superior vena cava (0.2%) [8]. No events involving the aortic arch or aorta have been reported.

The incidence of inadvertent subclavian artery puncture is reported at 3.7% due to its location [3], behind and slightly superior to the path of the subclavian vein [6]. Arterial punctures are directly proportional to the number of attempts made at catheterization, occurring at a rate of 50% after three attempts at cannulation according to studies by Lefrant *et al.* and Sznajder *et al.* [3, 3]. Critical care patients carry a higher rate of complication due to their altered anatomy, presence of localized edema, previous catheterizations, coagulation disorders, and urgent or emergent situation. Identified risk factors that contribute to the complication include obesity, previous surgery or radiotherapy to the area, history of catheterization at the same site, multiple cannulation attempts, and inexperienced operator [6].

Inadvertent subclavian artery cannulation brings risks such as occlusion, embolism, pseudo-aneurysm, dissection, perforation, exsanguination, and hemothorax [6, 6]. Morbidity and death may occur from inadvertent arterial puncture due to hemorrhage with blood collecting into the pleura and mediastinum from tracking. A catheter tip lying in the incorrect location will at least cause damage to the tunica intima, which may lead to dissection or even perforation of the vessel. Since the location of the subclavian artery is in an anatomically noncompressible area, it may require surgical intervention such as surgical repair with first rib excision, thoracotomy, percutaneous stent grafting, intraarterial balloon compression, and local percutaneous treatment [6].

In order to minimize the aforementioned complications, ultrasound guidance is highly recommended in subclavian catheterization because it allows for direct visualization of the anatomical structures as the needle traverses into the vessel. Two sonographic views are possible when utilizing this technique. The first view is through positioning the long footprint of the ultrasound probe perpendicular to the target vessel, to provide a short-axis view that permits the operator a midline orientation which allows for an out-of-plane approach. This does not offer the optimal ability to visually control the needle tip during cannulation because the needle artifact will only show the cross section of the needle, which may be any part of the needle shaft. The second view, the long-axis view, is obtained with the transducer and vessel axes in parallel, which will identify the target vessel along its length. This view allows the operator an in-plane approach, which permits direct and full visualization of both the needle tip and shaft during catheterization. However, this approach does not let the operator see both the artery and vein simultaneously as in the short-axis approach [11]. One of the most notable limitations of ultrasound is its dependence on the skill of the operator [1]. The subclavian cannulation approach had fallen out of favor through the years, possibly due to the difficulty in visualizing the vessels, which is underneath a reflective clavicle [11].

Another important process for minimizing the complications of subclavian catheterization is validation of placement after the procedure through either blood gas, transduction, and/or chest X-ray. Since the pulsatility and color of blood upon aspiration while doing the procedure do not distinguish between arterial and venous blood, confirmation through procurement of arterial blood gas and connecting the central line to a transducer ensures that the location of the catheter is not in the arterial circulation [3, 3, 14]. It is crucial to note that proper placement of the subclavian catheter tip should be just above the superior vena cava [3]. A subclavian catheter tip visualized left of the trachea raises a high probability of a malposition, and should be corrected immediately to prevent complications necessitating urgent interventional radiology or surgery consult to decide whether surgical or nonsurgical removal is required [13, 13].

## Conclusion

Subclavian vein cannulation is an alternative route in appropriately selected critically ill patients for central venous catheterization and carries a 0.3–12% risk of overall complications and a 3.7% risk of unintentionally puncturing the arterial vessel through the subclavian route. Utilization of real-time ultrasound guidance in this approach will allow direct visualization of needle insertion and adjacent anatomical structures, guidewire location and directionality, or can confirm pressure tracings, all of which can lead to decreased complications and improved cannulation success compared with the landmark technique. In addition, obtaining blood gas and/or attachment of the central line to a transducer after the procedure can be used to validate that arterial waveforms are not present if an ultrasound probe is not available.

This case report should raise awareness regarding the importance of real-time ultrasound in performing central line placements. Due to the reflection of the clavicle when ultrasound is used via the subclavian route, the landmark technique is often used and this option had fallen out of favor despite its importance. The ability to dampen artifactual reflections should be a consideration when future progress in ultrasound technology is achieved.

It is also worth mentioning the paramount importance of a postcatheterization roentgenogram to confirm proper cannulation and intervene early in the event of aberrant placement, as occurred in this patient. Clinicians and proceduralists should act immediately if a leftward direction of the catheter is seen, as this should raise suspicion of inadvertent catheter placement and requires urgent correction to prevent significant morbidity and mortality.

## Data Availability

Data sharing not applicable to this article as no datasets were generated or analyzed during the current study.
